# The Differential Expression of Kiss1, MMP9 and Angiogenic Regulators across the Feto-Maternal Interface of Healthy Human Pregnancies: Implications for Trophoblast Invasion and Vessel Development

**DOI:** 10.1371/journal.pone.0063574

**Published:** 2013-05-16

**Authors:** Mushi Matjila, Robert Millar, Zephne van der Spuy, Arieh Katz

**Affiliations:** 1 MRC/UCT Receptor Biology Unit, Division of Medical Biochemistry, Institute of Infectious Disease and Molecular Medicine, University of Cape Town, South Africa; 2 Department of Obstetrics and Gynaecology, Groote Schuur Hospital, Cape Town, South Africa; 3 Mammalian Research Institute, University of Pretoria, Pretoria, South Africa; 4 Centre for Integrative Physiology, University of Edinburgh, Edinburgh, Scotland; Virgen Macarena University Hospital, School of Medicine, Spain

## Abstract

Genes involved in invasion of trophoblast cells and angiogenesis are crucial in determining pregnancy outcome. We therefore studied expression profiles of these genes in both fetal and maternal tissues to enhance our understanding of feto-maternal dialogue. We investigated the expression of genes involved in trophoblast invasion, namely *Kiss1, Kiss1 Receptor (Kiss1R)* and *MMP9* as well as the expression of angiogenic ligands Vascular Endothelial Growth Factor-A (*VEGF-A)* and Prokineticin-1 (*PROK1*) and their respective receptors *(VEGFR1, VEGFR2* and *PROK1R*) across the feto-maternal interface of healthy human pregnancies. The placenta, placental bed and decidua parietalis were sampled at elective caesarean delivery. Real-time RT-PCR was used to investigate transcription, while immunohistochemistry and western blot analyses were utilized to study protein expression. We found that the expression of *Kiss1 (p<0.001), Kiss1R (p<0.05)* and *MMP9 (p<0.01)* were higher in the placenta compared to the placental bed and decidua parietalis. In contrast, the expression of *VEGF-A* was highest in the placental bed (*p<0.001*). While *VEGFR1* expression was highest in the placenta *(p<0.01),* the expression of *VEGFR2* was highest in the placental bed *(p<0.001).* Lastly, both *PROK1 (p<0.001)* and its receptor *PROK1R (p<0.001)* had highest expression in the placenta. Genes associated with trophoblast invasion were highly expressed in the placenta which could suggest that the influence on invasion capacity may largely be exercised at the fetal level. Furthermore, our findings on angiogenic gene expression profiles suggest that angiogenesis may be regulated by two distinct pathways with the *PROK1/PROK1R* system specifically mediating angiogenesis in the fetus and *VEGFA/VEGFR2* ligand-receptor pair predominantly mediating maternal angiogenesis.

## Introduction

Effective placentation is required for successful pregnancy outcome. During placentation, transformation of the spiral arteries from high resistance low capacity vessels to low resistance high capacity vessels is crucial for successful support of the conceptus and fetus. The lack of transformation of these spiral arteries is associated with poor pregnancy outcomes such as preeclampsia and intrauterine growth restriction (IUGR) [1], [2].

The transformation of spiral arteries is determined by adequate endovascular invasion of extravillous trophoblast (EVT) cells. Various factors determine the invasion capacity of these cells. Oxygen tension seems to have different effects on the invasive phenotypes of trophoblast cells depending on pregnancy duration [3], [4]. The effect of oxygen appears to be mediated via the actions of Hypoxia Inducible Factor (HIF) and Transforming Growth Factor β3 (TGFβ3) as well as alteration of Matrix Metalloprotease-9 (*MMP9*) expression [5], [6]. The ability of EVT cells to express gelatinases like MMP9 aids extracellular matrix degradation and enhances invasiveness. The expression of *MMP9* in particular, has been associated with high invasiveness, while that of Tissue Inhibitors of Metalloproteases (TIMPs) markedly reduces this invasive potential [7], [8].

*Kiss1* gene was initially discovered as a tumour metastasis suppressor gene of melanoma cells in nude mice without affecting their tumorigenicity [9]. This suppression of metastasis by *Kiss1* gene was subsequently demonstrated in various cancer cell-lines as well as in animal and human cancer experiments [10], [11], [12]. The *Kiss1* gene encodes peptides known appropriately as kisspeptins (kp). Posttranslational processing of the original *Kiss1* transcript results in kisspeptins of various lengths (kp-145, kp-54 also known as metastin, kp-14, kp-13 and the smallest cleavage product kp-10). Kisspeptins are natural ligands of G-Protein Coupled Receptor 54 (GPR54) also known as Kiss1 Receptor (Kiss-1R) [13], [14], [15].

The molecular machinery and pathways involved in tumour metastasis and invasion of extravillous trophoblast (EVT) cells into the maternal decidua are similar [16]. However the invasiveness of trophoblast cells is highly regulated temporally and spatially. The highest invasion is observed in the first trimester of pregnancy and geographically only extends as deep as the inner third of the myometrium. The clinical repercussions of “overinvasion” and “underinvasion” are placenta accreta as well as preeclampsia and IUGR, respectively. The former leads to massive obstetric haemorrhage secondary to retained placenta at the time of delivery while the latter (a consequence of maternal vascular maladaptation), contributes significantly to maternal and perinatal mortality, particularly in the developing world [17]. Kisspeptins limit the invasion of primary trophoblast cells and EVT cell-lines in vitro [18]. Furthermore up-regulation of *Kiss1* expression and down-regulation of *MMP9* gene expression have been associated with pregnancies complicated by preeclampsia and IUGR [19], [20].

Interplay between diverse factors affecting EVT invasion comprises a delicate equilibrium between pro-invasive and anti-invasive factors which is required for adequate invasion and a healthy gestation. Genes involved in angiogenic pathways appear to play a central role in achieving this equilibrium because ultimately the final common pathway to trophoblast invasion is spiral arteriolar transformation. In addition it has been demonstrated that the transformation of EVT cells from expressing an epithelial-type cell adhesion profile to an endothelial-type one enhances their invasiveness and subsequent transformation of spiral arteries [21].

The majority of factors involved in trophoblast invasion have mainly been studied in the fetal compartment of the feto-maternal interphase i.e. the placenta. Very few studies have examined both sides of this interface concomitantly. To better understand the molecular and cellular interactions involved in feto-maternal dialogue, both components of this interface should be studied in healthy pregnancies before even exploring pathological pregnancies.

In this study, we examined the gene expression profiles of *Kiss1* and its cognate receptor *GPR54* (*Kiss1R*) in the placenta, placental bed and decidua parietalis. In addition we investigated the expression profiles of gelatinase B (*MMP9)* which is involved in extracellular matrix degradation across the three feto-maternal tissue compartments. Lastly, the expression of genes involved in angiogenesis, namely Vascular Endothelial Growth Factor A (*VEGF-A*), Prokineticin-1 (*PROK1*) also known as Endocrine Gland-specific VEGF (*EG-VEGF*) and their respective receptors (*VEGFR1, VEGFR2*) and *PROK1R* were investigated in the placenta, placental bed and decidua parietalis.

## Materials and Methods

### Ethics Statement

The study was approved by the Human Research Ethics Committee of the Faculty of Health Sciences, University of Cape Town (REF: 080/2008). Written informed consent was obtained from all patients and the research was conducted according to the ethical principles of the Helsinki Declaration [22]. Patients were counselled prior to the day of their elective procedure in their preferred language of choice. They were presented with Patient Information Leaflets (PILs) which provided the details of the study. The capacity of the patients to comprehend this information was always assessed and where there was doubt about comprehension, consent for study participation was not taken. Within the consent form, there was a section reassuring the patients that their decision involving participation in the study would not in any way compromise their further management and care. The patients were at liberty to make this choice without any force or coercion.

### Study Participants

All patients recruited had elective caesarean section deliveries in the absence of labour. The inclusion criteria for the study were healthy patients undergoing elective caesarean section for the indication of previous caesarean section or fetal malpresentation. Patients in labour, with a background of two previous caesarean sections, with pregnancy-related complications such as gestational diabetes, gestational hypertention, placenta praevia, preeclampsia, intrauterine growth restriction as well as patients with underlying medical disorders were excluded from the study. Patients were recruited from Groote Schuur and Mowbray Maternity Hospitals in Cape Town, South Africa.

### Tissue Sampling and Specimen Collection

The placenta and placental bed were sampled at elective caesarean section using a previously described technique [23]. Briefly, prior to delivery of the placenta, apposing sections of the placenta and placental bed were marked by placing a needle and suture from the placenta and traversing the uterine wall. A core of the placental tissue around the suture in apposition to the maternal surface (i.e which contains the maternal end of the placenta) was then sampled and the rest of the placenta delivered. The section of the placental bed where the sampled placenta was resident could then be identified with the aid of the remaining suture and was subsequently biopsied. The adequacy of the sampled placental bed was confirmed by an independent histopathologist (L.M). In addition, the decidua parietalis which has no involvement with placentation was sampled. Collected tissue samples were divided into three portions. One portion was collected for RNA extraction, while the second and third portions were utilised for protein extraction and preparation of wax-embedded tissue blocks respectively.

### RNA Extraction

1 ml of TRIzol® reagent (Invitrogen™) was used per 50 mg of tissue. Tissue was homogenised on ice with a Tissue Ruptor® (Qiagen). The homogenate was centrifuged using the Eppindorff 5804 R machine (15294 g at 4°C for 15 minutes (mins)) and the supernatant was collected and added to 200 µl of ice-cold BCP (1-Bromo-3-Chloro-Propane (Sigma). The mixture was shaken for 15 seconds (secs) and kept on ice for 10 mins. The solution was centrifuged again and 500 µl of propanol was added to precipitate the RNA. The RNA pellets were washed in 75% ethanol, air dried and re-suspended in DEPC- treated water.

### cDNA Synthesis

RNA was reverse transcribed with Multiscribe Reverse Transcription reagents (Applied Biosystems). For a 20 µl reverse transcription reaction, the following were used; DEPC-treated H_2_O (3.7 µl), 10x RT Buffer (2 µl), MgCl_2_ (4.4 µl), dNTPs (4 µl), Random Hexamers(1 µl), RNAse Inhibitor (0.4 µl), Reverse Transcriptase (0.5 µl) and (2 µl) of RNA. The ABI GeneAmp® 2700 Thermal Cycler was used and the cycling parameters were 25°C (10 mins), 4°C (10 mins), 48°C (45 mins) and 95°C (5 mins).

### Real-Time PCR

Gene expression studies were conducted using the ABI 7900 RT-PCR instrument (Applied Biosystems). The standard thermal cycling protocol was conducted as follows: 50°C for 2 mins, 95°C for 10 mins and 40 cycles of (95°C for 95 secs and 60°C for 1 min). The Genebank accession numbers of the genes investigated are as follows: *KiSS1* (NM_002256), *KiSS1R* (NM_032551), *MMP9* (NM_004994), *VEGFA* (NM_001171623), *VEGFR1 (FLT)* (NM_002019), *VEGFR2 (KDR)* (NM_002253), *PROK1* (NM_032414), *PROKR1* (NM_138964). Primer/Probe pairs corresponding to these genes (Sigma® Pharmaceuticals) were utilised (Table 1) in conjunction with the Taqman® Mastermix. All RT-PCR gene expression data is presented as Means ± SEM and gene expression was relative to 18 s ribosomal RNA (internal control) and reference cDNA.

**Table 1:** Primers and Probe Sequences used for Real-Time (RT) PCR Reactions.

1GPR54 Forward Primer	5'-GGTGCTGGGCGACTTCAT-3'
GPR54 Reverse Primer	5'-CACACTCATGGCGGTCAGAGT-3'
GPR54 Probe	5'-[FAM]-TGCAAGTTCGTCAACTACATCCAGCAGG-[TAMRA]-3'
2Kiss1 Forward Primer	5'-GGCAAGCCTCAAGGCACTT-3'
Kiss1 Reverse Primer	5'-GGAAAAGCAGTAGCTGCCAAGA-3'
Kiss1 Probe	5'-[FAM]-TGCCTCTTCTCACCAAGATGAACTCACTGG-[TAMRA]-3'
3PROK1 Forward Primer	5'-GTG CCA CCC CGG CAG-3'
PROK1 Reverse Primer	5'-AGC AAG GAC AGG TGT GGT GC-3'
PROK1 Probe	5'-[FAM]-ACA AGG TCC CCT TCT TCA GGA AAC GCA-[TAMRA]-3'
4PROKR1 Forward Primer	5'-TCT TAC AAT GGC GGT AAG TCC A-3'
PROKR1 Reverse Primer	5'-CTC TTC GGT GGC AGG CAT-3'
PROKR1 Probe	5'-[FAM]-TGC AGA CCT GGA CCT CAA GAC AAT TGG-[TAMRA]-3'
5VEGF Forward Primer	5'-TAC CTC CAC CAT GCC AAG TG-3'
VEGF Reverse Primer	5'-TAG CTG CGC TGA TAG ACA TCC A-3'
VEGF Probe	5'-[FAM]-ACT TCG TGA TGA TTC TGC CCT CCTCCT T-[TAMRA]-3'
6MMP9 Forward Primer	5'-GGCCACTACTGTGCCTTTGAG-3'
MMP9 Reverse Primer	5'-GATGGCGTCGAAGATGTTCAC-3'
MMP9 Probe	5'-[FAM]-TTGCAGGCATCGTCCACCGG-[TAMRA]-3'
7VEGFR2 Forward Primer	5'-TTA CAG CTT CCA AGT GGC TAA GG-3'
VEGFR2 Reverse Primer	5'-ATT TTA ACC ACG TTC TTC TCC GAT AA-3'
VEGFR2 Probe	5'-[FAM]-CTT GGC ATC GCG AAA GTG TAT CCA CA-[TAMRA]-3'
8VEGFR1 Forward Primer	5'-ATG TGC CAA ATG GGT TTC ATG T-3'
VEGFR1 Reverse Primer	5'-ACT TGT TAA CTG TGC AAG ACA GTT TCA-3'
VEGFR1 Probe	5'-[FAM]-CTC TCC TTC CGT CGG CAT TTT TTC CAA-[TAMRA]-3'

1 G-Protein Coupled Receptor 54 (Kiss1 Receptor)

2 Metastasis Suppressor Gene (Kiss1)

3 Prokineticin1

4 Prokineticin1- Receptor

5 Vascular Endothelial Growth Factor

6 Matrixmetalloprotease-9

7 Vascular Endothelial Growth Factor Receptor 2

8 Vascular Endothelial Growth Factor Receptor 1

### Immunohistochemistry

Tissue blocks were sectioned and fixed to slides (Histobond®) with the placenta, placental bed and decidua parietalis fixed across one slide and stained together.

#### Kisspeptin immunostaining

Slides were de-waxed in xylene and rehydrated in decreasing concentrations of ethanol. Antigen retrieval was done by pressure cooking the slides in 0.01 M Citrate Buffer. The slides were then blocked in 10% Methanol Hydrogen Peroxide for 30 mins, washed in water and then TBS (Tris-Buffered Saline). Normal Donkey Serum (NDS) was used for blocking followed by incubation with Sheep anti-kisspeptin54 (GQ2) antibody (1∶1500) (provided by Professor Bloom’s laboratory) overnight at 4°C. Thereafter slides were washed in TBS (2×5 mins) and incubated for 30 mins with secondary Donkey anti-Sheep Peroxidase antibody (Jackson Immunoresearch) (1∶750) (1.8 mg/ml).

The slides were washed again in TBS (3×5 mins) and incubated for 10 mins with Tyramide Cyanine-5 (Perkin Elmer) (1∶50). Thereafter slides were washed in TBS (2×5 mins) and microwaved in citrate buffer for 2.5 mins and incubated in hot buffer for 30 mins. They were then washed in TBS and incubated with Mouse anti-α-Smooth Muscle Actin antibody (Sigma Aldrich) (1∶5000) for 1 hour. After washing in TBS, secondary Donkey anti-Mouse-555 (DAM-555) (1∶200) (2 mg/ml) (Invitrogen) antibody was applied for 30 mins and washed off with TBS. Nuclear stain DAPI 405, (1∶1000 in PBS)**,** was applied for 10 mins at room temperature and washed in TBS. Slides were then mounted with Permaflour® and viewed with Zeiss LSM 710 META confocal microscope.

#### GPR54 immunostaining

The same protocol as above was followed except no antigen retrieval was required and Normal Goat Serum was used for blocking. Primary Rabbit anti-GPR54 (R2 1213) custom manufactured by EZ Biolabs (1∶1600) was used. Pre-immune serum was utilised as negative control. Goat anti-Rabbit Peroxidase (1∶200) (Dako) was used as secondary antibody followed by Tyramide-546 (Cyanine-3) (Perkin Elmer). Sytox Green (1∶1000 in TBS**)** was used to stain the nuclei.

#### MMP9 immunostaining

The slides were de-waxed and rehydrated. Heat-mediated antigen retrieval in 0.01 M citrate buffer was followed by quenching endogenous peroxide with 30% methanol hydrogen peroxide. Blocking to prevent non-specific antibody binding was done with NDS and primary rabbit anti-MMP9 antibody (ab38898 (1 mg/ml)) (1∶200) from Abcam® was applied overnight. Donkey Anti-Rabbit-555 (1∶1000) (A31572 from Invitrogen) was used as secondary antibody followed by DAPI staining for nuclei.

#### PROK1 immunostaining

The slides were prepared as above with antigen retrieval, blocked with NDS and incubated with primary Rabbit anti-PROK1 antibody (Abcam®) (1∶50) in NDS. Donkey Anti-Rabbit-555 (DAR-555) (1∶1000) (2 mg/ml) from Invitrogen was used as secondary antibody and the nuclei were stained with DAPI (1∶1000 in PBS).

#### VEGF-A immunostaining

Slides were de-waxed, rehydrated, antigen retrieved and bathed in hydrogen peroxide for 30 mins. Blocking was done with NDS. Rabbit Anti-VEGF A antibody (VEGF (sc-152) (1∶50) (200 µg/ml) (Santa Cruz) and DAR-555 (1∶1000) were used as primary and secondary antibody respectively. The nuclei were stained with DAPI.

#### VEGF-A and pancytokeratin Co-Immunostaining

Slides were de-waxed, rehydrated, antigen retrieved and blocked with methanol hydrogen peroxide. NDS was used for blocking. Rabbit anti-VEGF-A (1∶50) and Mouse anti-Pancytokeratin (1∶15000) (Sigma) were applied to the slides and incubated overnight. DAR-555 (1∶1000) and Goat Anti-Mouse Alexa-488 (1∶500) in NDS were used as secondary antibodies. DAPI was used to stain the nuclei.

#### VEGFR1 and VEGFR2 Co-Immunostaining

The slides were de-waxed and rehydrated. Heat-mediated antigen retrieval was done in 0.01 M Citrate buffer followed by quenching of endogenous tissue peroxidase activity with 30% methanol hydrogen peroxide for 30 minutes. NDS was employed to block non-specific antibody binding. The slides were incubated simultaneously with mouse anti-VEGFR1 (ab9540) and rabbit anti-VEGFR2 antibody (ab2349) from Abcam®. Goat Anti-Mouse Alexa-488 and Donkey Anti-Rabbit-555 were used concurrently as secondary antibodies. DAPI was utilised for nuclei staining.

### Protein Extraction and Quantification

RIPA lysis buffer (25 mM Tris HCl pH 7.6, 150 mM NaCl, 1% Triton X, 1% sodium deoxycholate and 0.1% SDS) was used for tissue lysis. 4 M Urea and protease inhibitor tablets (Roche®) were added to the buffer. The tissue was incubated on ice for 30 mins, then poured into a mortar, snap-frozen with liquid nitrogen and crushed into fine powder using a pestle. The powder was collected in 2 ml epindorff tubes and centrifuged at 12000 rpm for 15 mins @ 4°C. The supernatant was collected and protein concentration quantified using the Pierce BCA Protein Assay Kit (Thermoscientific).

### Western Blot Analysis

20 µg of protein/well was loaded after denaturing in loading buffer at 95°C for 5 mins. The proteins were separated on a 15% SDS-PAGE gel run at 110 V for 80 mins. The gel was then blotted onto a 0.45 µm PVDF membrane (Amersham Hybond-P) using Novex® Semi-dry Blotter (Invitrogen). The membranes were blocked at room temperature for 1hour in 3% BSA and then incubated at 4°C overnight with Rabbit anti-VEGF-A antibody (sc-152) (1∶50). For negative control the membrane was pre-incubated with a mixture of anti-VEGF-A and VEGF-A blocking peptide (Abcam®). After washing, Goat anti-Rabbit Peroxidase (1∶5000) was applied and the blot was visualised using a film (Amersham Hyperfilm™ ECL- GE Healthcare Limited). Beta-Actin (β-Actin) was used as loading control.

### Statistical Analysis

Graphpad 5 Prism software (version 5.00 for Windows, GraphPad Software, San Diego California USA, www.graphpad.com) was utilised for statistical analysis with One-Way ANOVA followed by Dunn’s Multiple Comparison’s Test. P<0.05 conferred statistical significance.

## Results

### Clinical Data

Twenty-eight patients with healthy pregnancies were included in the study. The indications for caesarean section were a previous caesarean section in 23 patients (82.14%) and malpresentations in five (17.86%) patients. The clinical parameters are presented as means with standard error of the means (Mean ± SEM) (Table 2).

**Table 2:** Clinical Data.

Age (years)	Gravidity	Parity	Gestation (weeks)
28.47±0.87	2.433±0.18	1.1±0.16	38.03±0.06

Table 2 depicts patients clinical data presented as means with standard error of the means (means ±SEM) showing Age (in years), Parity (number of pregnancies that have reached viability), Gravidity (number of pregnancies irrespective of viability) and Gestation (duration of pregnancy in weeks).

### *Kiss1* and *GPR54* Expression are Highest in the Placenta

**Figure 1:**
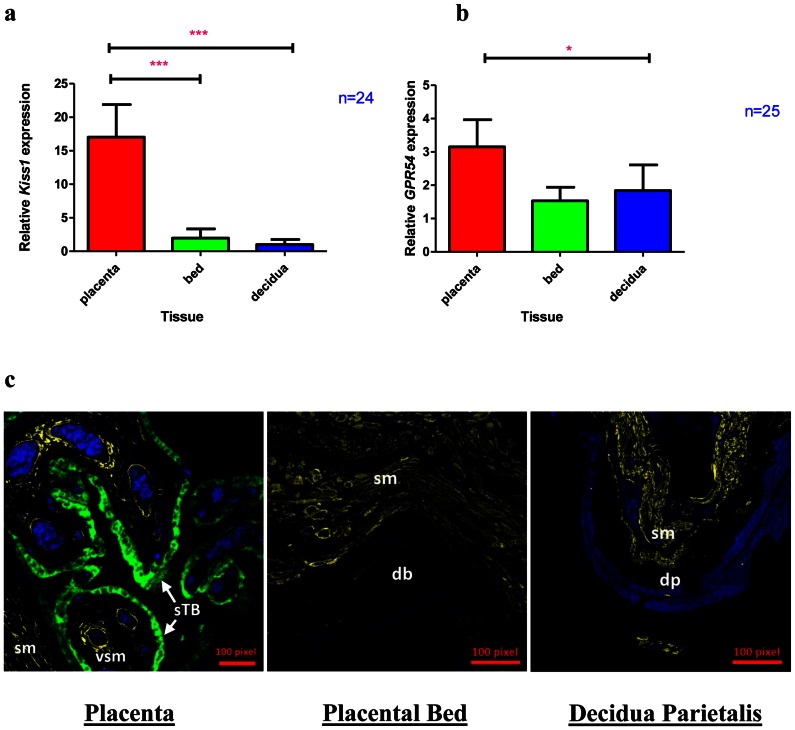
Kiss1 and GPR54 (Kiss1 R) expression in the Placenta, Placental bed and Decidua parietalis. Real-time PCR (Means ± SEM) showing *Kiss1* (Fig. 1a) and *GPR54* (Fig. 1b) expression in the three feto-maternal compartments. (***) and (*) signify p<0.001 and P<0.05 respectively. Fig. 1c shows kisspeptin immunostaining in the Placenta, Placental Bed and Decidua Parietalis, with kisspeptin protein expression (green fluorescent staining) in the placental syncitiotrophoblast (sTB) layer. Smooth Muscle Actin (yellow) was used as a positive control for slide staining and can be seen staining vascular smooth muscle (vsm) and smooth muscle (sm). The decidua basalis (db) and decidua parietalis (dp) can be seen in the placental bed and decidual sections respectively. Kisspeptin staining is negative in the placental bed and decidua parietalis. The nuclei were stained with Dapi (blue).

The expression of *Kiss1* and *GPR54* genes in the placenta, placental bed and decidua was determined by RT-PCR (Fig. 1). There was seven times more *Kiss1* expression in the placenta compared to the placental bed and eighteen times more *Kiss1* expression in the placenta compared to the decidua parietalis (p<0.001). However there was no significant difference in *Kiss1* gene expression between the placental bed and the decidua parietalis. The gene expression level of *Kiss1R* was modestly higher (1.6 times) in the placenta compared the placental bed and decidua parietalis (Fig. 1b) and its expression in the placenta was much lower compared to that of *Kiss1*.

**Figure 2:**
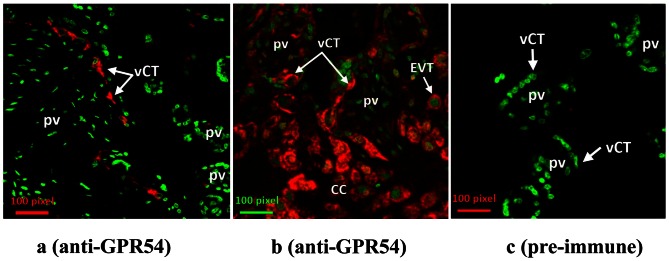
GPR54 (Kiss1R) immunostaining in the Placenta. Fig. 2 (a–c) demonstrate GPR54 immunohistochemistry with individual villous cytotrophoblast cell staining for GPR 54 (red) in the placental villus (pv) (Fig. 2a). Fig. 2b shows GPR54 (red) immunostaining of villous cytotrophoblasts (vCT), cytotrophoblast columns (CC) and extravillous trophoblast cells (EVTs). The placenta is stained with GPR54 pre-immune serum (negative control) in Fig. 2c. The nuclei are stained with Sytox Green (green).

Immunohistochemical studies detected kisspeptin protein expression only in the placenta (Fig. 1c). Kiss1R (GPR54) protein was expressed in the placenta and localized to the villous syncitiotrophoblast and cytotrophoblast cell layers as well as the extravillous trophoblast cell population (Fig. 2a–c). No GPR54 immunostaining was observed in the placental bed and decidua basalis (data not shown).

### *MMP9* Transcript Expression is Highest in the Placenta

**Figure 3:**
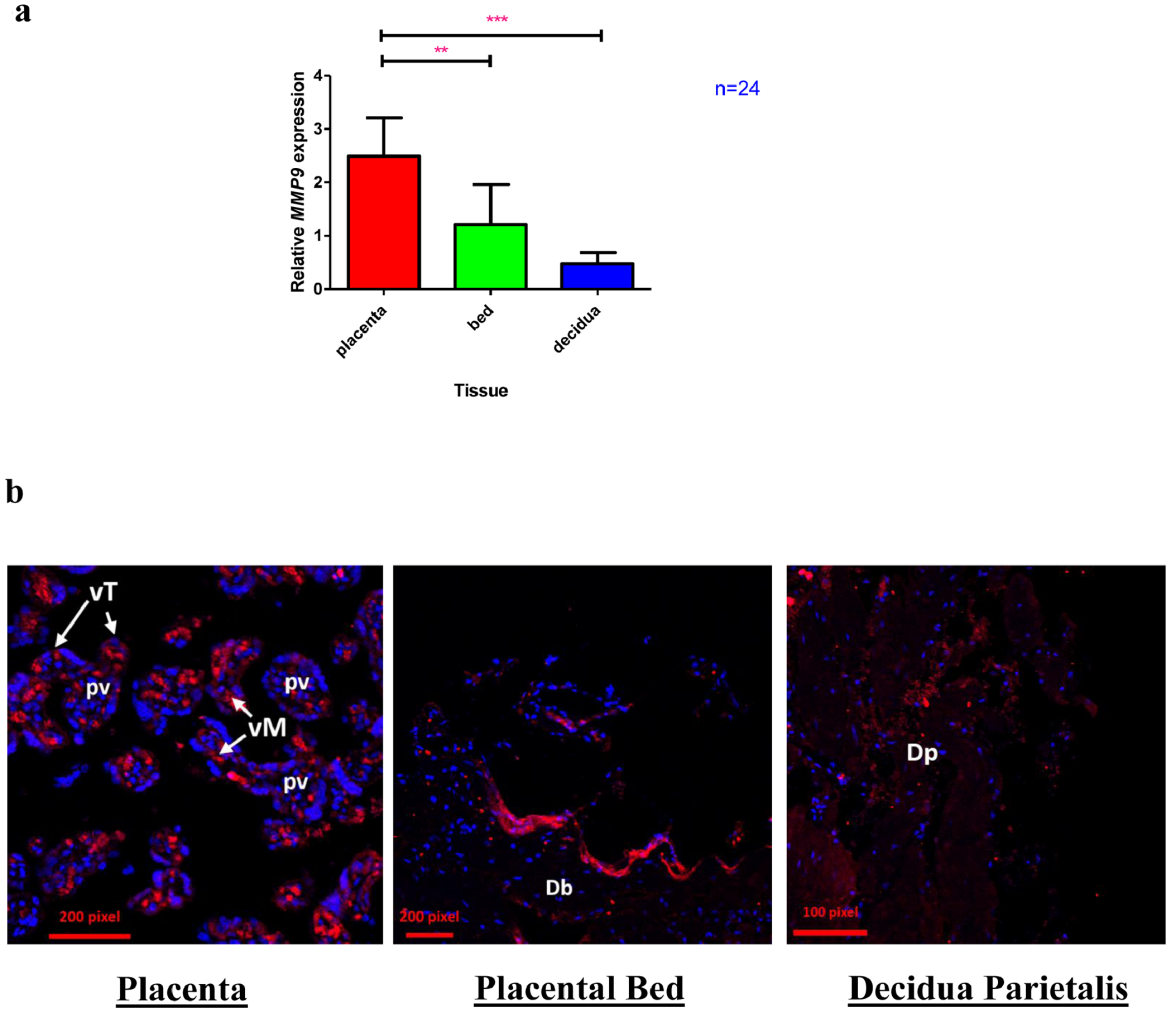
*MMP9* expression in the Placenta, Placental Bed and Decidua Parietalis.

Next we determined the *MMP9* mRNA expression in the three feto-maternal tissues. We found that *MMP9* mRNA expression was at least two-fold higher in the placenta compared to the placental bed and four-fold higher in the placenta compared to the decidua parietalis (Fig. 3a). Immunohistochemistry was used to localize MMP9 protein in the placenta, placental bed and decidua parietalis (Fig. 3b). MMP9 protein was localized in the villous trophoblast cells and mesenchyme of the placenta while in the placental bed the epithelial border of the decidua basalis stained positive for MMP9. There was minimal MMP9 staining in the decidua parietalis.

### *VEGF-A* Gene and Protein Expression are Highest in the Placental Bed

**Figure 4:**
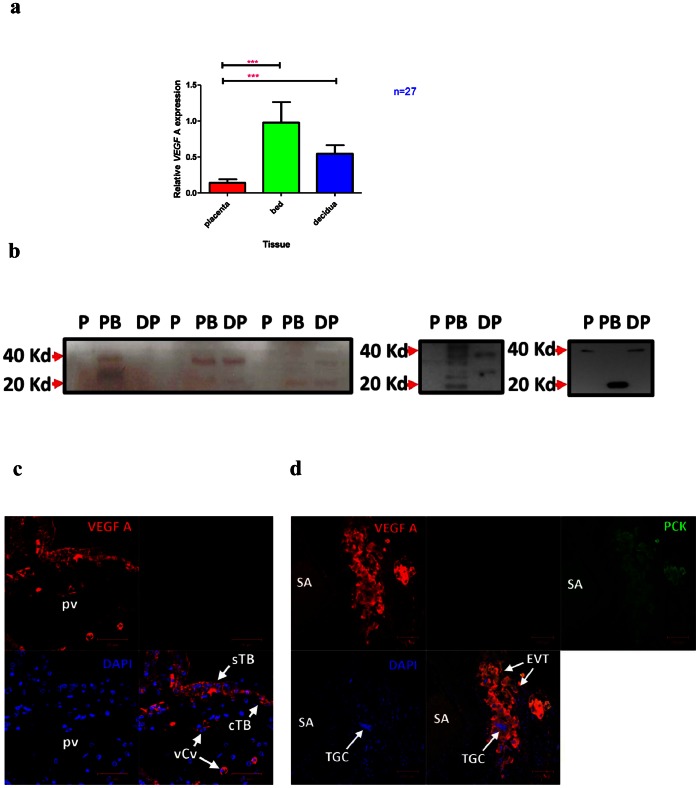
The expression of *VEGF-A* in the Placenta, Placental Bed and Decidua Parietalis. Real-time PCR (Means ± SEM) showing mRNA expression of *VEGF-A* (Fig. 4a), in the three feto-maternal compartments. (***) and (**) signify p<0.001 and p<0.01 respectively. The highest *VEGF-A* mRNA is expressed in the placental bed. Fig. 4b shows VEGF-A Western Blot Analysis of the Placenta, Placental Bed and Decidua Parietalis obtained from different patients. Bands are present in both the maternal Placental Bed (PB) and Decidua Parietalis (DP). Bands between 20 and 40 Kd represent the various VEGF-A isoforms. The molecular weight of the VEGF is 20 Kd (Kilodaltons) while that of the homodimer is 40 Kd. Figure 4c demonstrates VEGF-A immunostaining in the placenta with VEGF-A protein expression (red) in both the syncytiotrophoblast (sTB) and cytotrophoblast (cTB) cell layers of the placental villus (pv). VEGF-A staining is also observed in the endothelial cells of villous capillary vessels (vCv). DAPI (blue) is used to stain the nuclei. Fig. 4d shows co-localisation of VEGF-A (red) and Pancytokeratin (PCK) (green) in a cluster of Extravillous Trophoblast (EVT) cells staining yellowish-orange. Multinucleated Trophoblast Giant Cells (TGCs) can be seen amongst the EVTs. The lumen of a spiral artery (SA) containing red blood cells can be noticed on the left. DAPI (blue) was used to stain nuclei. The nuclei of the TGCs are different in morphology and size in comparison to those of the surrounding cells.

*VEGF-A* gene expression was 7-fold higher in the placental bed compared to the placenta (p< 0.001) and 1.6-fold higher in the placental bed compared to the decidua parietalis (Fig. 4a). Western Blot analysis confirmed RT-PCR findings showing more VEGF-A protein expression in the placental bed and decidua parietalis compared to the placenta (Fig. 4b). Immunostaining of placental sections showed mild VEGF-A staining of the syncytiotrophoblast, cytotrophoblast as well as staining of villous capillary endothelial cells (Fig. 4c). On the other hand, we observed strong VEGF-A immunostaining in the placental bed sections relative to the placenta and decidua parietalis (not shown). Immuno-colocalization of VEGF-A and Pancytokeratin (a marker for trophoblast cells) revealed that placental bed VEGF-A expression was predominantly from extravillous trophoblast cells and trophoblast giant cells (Fig. 4d).

### *VEGFR1* and *VEGFR2* Transcripts and Protein are Differentially Expressed Across the Feto-maternal Interface

**Figure 5:**
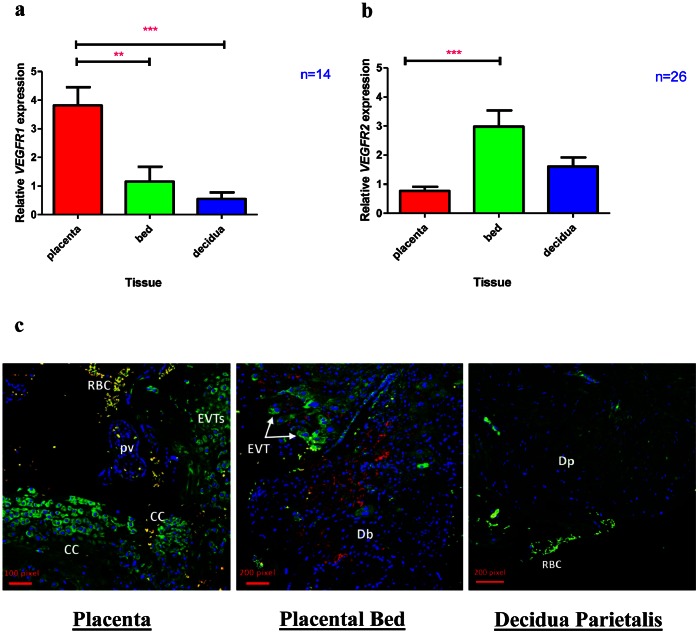
The Expression of VEGF Receptors in the three feto-maternal compartments. Fig. 5a demonstrates *VEGFR1* and Fig. 5b shows *VEGFR2* mRNA expression in the placenta, placental bed and decidua parietalis. The placenta has highest *VEGFR1* mRNA expression (Fig. 5a) while the placental bed has highest expression of *VEGFR2* mRNA (Fig. 5b). Figure 5c demonstrates VEGFR1 and VEGFR2 co-immunostaining in the placenta and placental bed and decidua parietalis. In the placental section VEGFR1 (green) staining can be seen in the cytotrophoblast columns (CC) and extravillous trophoblast (EVT). No VEGFR2 staining (red) was observed in the placental sections. Red blood cells (RBCs) auto-flouresce with both green and red dyes and can be seen (yellow) in the image. In the placental bed section both VEGFR1 (green) and VEGFR2 (red) staining were detected. VEGFR1 staining was observed in the interstitial extravillous trophoblast (EVT) of the decidua basalis while VEGFR2 staining was limited to the decidual stroma. There was comparatively minimal VEGFR1 and VEGFR2 staining in the decidua parietalis (Dp).

VEGF-A mediates its effects via interaction with a number of its receptors. We examined the mRNA expression of receptors VEGF Receptor 1 (*VEGFR1)* and VEGF Receptor 2 (*VEGFR2)* in the feto-maternal tissues. *VEGFR1* also known as *FLT* (fms-like tyrosine kinase) expression was highest in the placenta (3-fold higher expression in the placenta compared to the placental bed and 7-fold higher expression in the placenta compared to the decidua parietalis) (Fig. 5a). In contrast, *VEGFR2* also called *KDR* (Kinase Domain Receptor) mRNA expression was highest in the placental bed (almost 4-fold more expression compared to the placenta) (Fig. 5b). Using immunohistochemical co-localisation of *VEGFR1* and *VEGFR2,* we found that *VEGFR1* was expressed by the cytotrophoblast columns (CC) as well as extravillous cytotrophoblast (EVT) in the placental sections and interstitial trophoblast cells in the placental bed. There was minimal staining of *VEGFR2* in the placenta but the most staining for this receptor was demonstrated in the placental bed. *VEGFR2* staining in the placental bed was neither from interstitial trophoblast nor decidual macrophages as verified with CD68 staining (data not shown) but was limited to the decidual stroma.

### *PROK1* and *PROK1R* Transcript and Protein Expression are Highest in the Placenta

**Figure 6:**
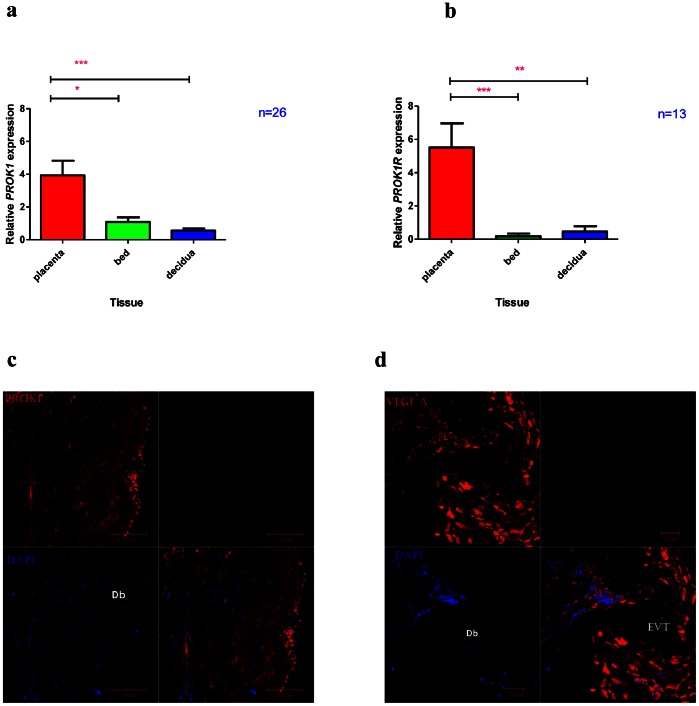
*PROK1* and *PROK1R* expression in the Placenta, Placental Bed and Decidua Parietalis. Real-time PCR (Means ± SEM) showing *PROK1* (Fig. 6a) and *PROK1R* (Fig. 6b) mRNA expression in the three feto-maternal compartments. (***), (**) and (*) signify p<0.001, p<0.01 and p<0.05 respectively. Fig. 6c and 6d show comparative PROK1 and VEGF-A staining in the placental bed respectively. There is minimal PROK1 staining (red) in the placental bed (Fig. 6c). In comparison Fig. 6d demonstrates strong VEGF-A immunostaining (red) of extravillous trophoblast cells (EVTs) in the placental bed. Dapi (blue) was used to stain the nuclei.

**Figure 7:**
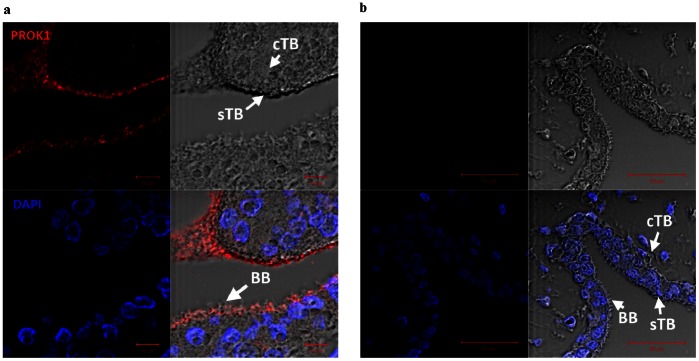
PROK 1 immunostaining in placental tissue. PROK1 immunostaining (red) can be seen most abundantly towards the microvillous brush border (BB) of the syncytiotrophoblast layer in Fig. 7a. The nuclei were stained with DAPI (blue). Morphological detail of the villous cytotrophoblast (cTB) and syncytiotrophoblast (sTB) cells are indicated (top right). The PROK1 primary antibody was omitted in Fig. 7b (negative control) and no PROK1 immunostaining is demonstrable (top left and bottom right).

The gene expression of both Prokineticin 1 (*PROK1*) (otherwise known as Endocrine Gland specific VEGF (EG-VEGF)) and its receptor *PROK1R* were highest in the placenta compared to the placental bed and decidua parietalis (Fig. 6a and 6b). Minimal PROK1 immunostaining was observed in the extravillous trophoblast cells (EVTs) of placental bed sections (Fig. 6c). In contrast VEGF-A immunostaining was strong in the interstitial EVTs of the placental bed (Fig. 6d). Immunostaining of the placental sections localized PROK1 protein to the microvillous border of the placental syncytiotrophoblast layer (Fig. 7).

## Discussion

Understanding normal feto-maternal dialogue is the first step in deciphering pregnancy-related complications and can be valuable in optimizing assisted reproductive technology. In this paper we investigated the expression profiles of genes involved in trophoblast invasion and angiogenesis which are both essential for placentation. We examined the expression of *Kiss1*, *VEGF-A*, *PROK1* and their respective receptors *GPR54*, *VEGFR1, VEGFR2* and *PROK1R* as well as the expression of MMP9 in the placenta, placental bed and decidua parietalis. The study was conducted in a population of young healthy patients mostly in their second pregnancies with no pregnancy-related complications or chronic medical illnesses.

**Figure 8:**
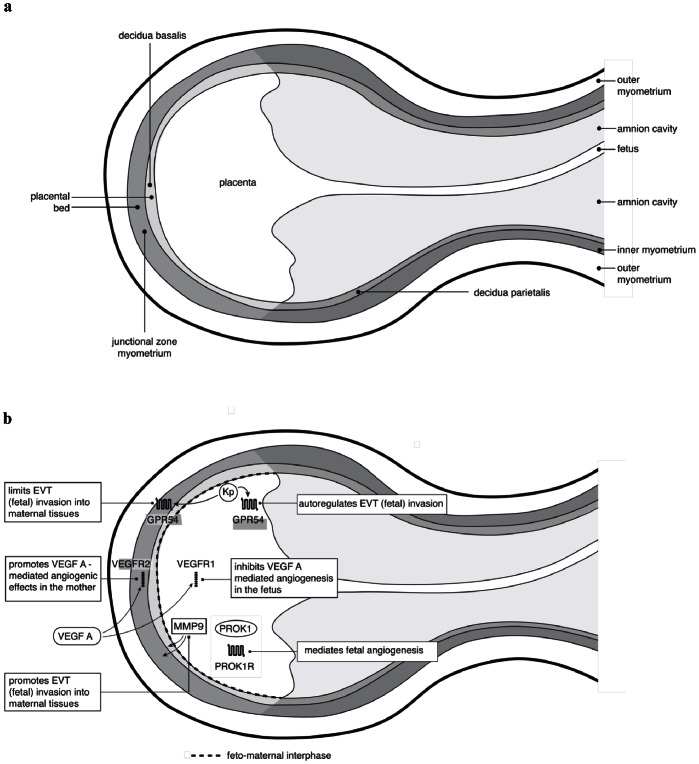
Schematic representation of the feto-maternal interface. Fig. 8a represents a longitudinal section of the anatomy of the feto-maternal interface while the putative molecular interactions at this interface are depicted in Fig. 8b.

Kisspeptin via its receptor, GPR54 has previously been shown to play an important role in tumour metastasis suppression and trophoblast invasion. To our knowledge, *Kiss1*, kisspeptin and *GPR54* have never been investigated across feto-maternal tissues in particular the maternal placental bed and decidua parietalis. We observed strikingly high *Kiss1* gene expression in the placenta and virtually no kisspeptin (protein) expression in the maternal tissues (placental bed and decidua parietalis). In contrast, *GPR54* gene expression in the maternal tissues was only 50% less than that expressed in the placenta. Kisspeptin and GPR54 have previously been shown to inhibit trophoblast invasion in an autocrine/paracrine manner in primary human trophoblasts [18]. The maternal expression of GPR54 may therefore play a role in limiting excessive invasion of trophoblast cells in the maternal tissues while the high placental kisspeptin and GPR54 expression may be involved in fetal autoregulation of maternal tissues (Fig. 8). This may suggest that the control of trophoblast invasion via the kisspeptin/GPR54 pathway is primarily exercised at the fetal level and is not only restricted to the first trimester but maintained to term. Kisspeptin could therefore have a role in the maintenance of placental invasive homeostasis until term.

We further demonstrated high *MMP9* expression in the placenta which likely enhances the ability of the fetus to invade the maternal tissues via degradation of extracellular matrix. Kisspeptin downregulates *MMP9* expression in various cancers [24], [25] and this may be the mechanism by which kisspeptin inhibits trophoblast invasion. Furthermore, *Kiss1* has been shown to reduce *MMP9* transcription by interfering with NFκβ binding to the *MMP9* promoter in a cell line model [25]. Downregulation of *MMP9* expression is associated with poor fetal growth and maternal preeclampsia [20], [26], [27] most likely by a mechanism involving limited trophoblast invasion and poor transformation of maternal spiral arteries. These data demonstrate the importance of the expression of gelatinases (amongst other proteases) by the fetal compartment in sustaining healthy pregnancies.

In our study *VEGF-A* expression was highest in the maternal compartment (placental bed), yet the focus in the literature is largely on *VEGF* expression in the placentae of pathological pregnancies [28], [29], [30], [31], [32], [33]. Consistent with our RT-PCR findings, we found VEGF-A protein expression to be highest in the placental bed and decidua parietalis. We have further demonstrated that the extravillous trophoblast (EVTs) and Trophoblast Giant Cells (TGCs) express VEGF-A protein in the placental bed. A study using in-situ hybridization showed that macrophages in the maternal decidua [34] expressed the highest VEGF-A mRNA however our studies detecting VEGF-A protein clearly showed localization to EVTs and minimal VEGF-A localization to macrophages. Future studies investigating pregnancy pathologies should therefore focus on VEGF-A expression changes in the placental bed rather than in the placenta.

When examining VEGF receptors, we found the expression of *VEGFR1* to be highest in the placenta while that of *VEGFR2* was highest in the maternal placental bed and decidua. *VEGFR1* also known as the “decoy” receptor, is thought to bind to VEGF-A and thus scavenge and reduce the amount of bioavailable VEGF-A to bind to VEGFR-2 (which mediates its angiogenic and mitogenic effects) [35], [36]. We found low VEGF-A and high VEGFR1 in the placenta. VEGF-A may (via interaction with VEGFR1) be suppressed in the placenta while VEGFR2 mediates the angiogenic and mitogenic effects of VEGF-A in the maternal placental bed. Interestingly immunohistochemical stainining for VEGFR1 was strong in extravillous trophoblast (cytotrophoblast columns in the placenta and interstitial trophoblast in the placental bed). Could this possibly be a mechanism by which these invading “fetal” cells suppress maternal angiogenesis in favour of their own angiogenic enhancement? Previously high expression of *VEGFR1* both in the placenta and circulation (sFLT1) of pregnant women has been reported and was thought to regulate the action of VEGF-A in successful pregnancies [37]. On the other hand, both VEGF-A and VEGFR2 protein expression are reduced in the placentae of first trimester miscarriages compared to healthy controls [38]. Furthermore both VEGF as well as VEGF receptor knockout mice result in embryonic lethality [39], [40], [41]. This indicates the importance of VEGF-mediated vasculogenesis and angiogenesis in early pregnancy. Our findings suggest that VEGF-A may be more important in the placental bed than the placenta in healthy human pregnancies at term. This would imply the potential role of a different angiogenic factor other than VEGF in maintaining placental angiogenesis after de novo vasculogenesis is mediated by VEGF in early pregnancy.

Our study has demonstrated using RT-PCR that both the mRNA expression of *PROK1* and *PROK1R* were highest in the placenta while that of *VEGF-A* was highest in the placental bed. PROK1 and its cognate receptor PROK1R have recently been shown to be important tissue-specific role players in the angiogenesis of steroidogenic organs like the ovary, testis, adrenal and the placenta [42]. The action of PROK1 is thought to be complementary to that of VEGF in these tissues. PROK1 has also been shown to be important in enhancing feto-maternal dialogue in receptive endometrium [43] and thus likely to be important in early healthy pregnancies.

By employing immunohistochemical analysis on first trimester placental tissue another study demonstrated the differential expression of VEGF and PROK1 in the placenta, with the former being expressed in the cytotrophoblast and EVT cells whilst the latter was distinctly expressed in the syncytiotrophoblast [44]. Of importance, this study found no PROK1 staining in the extravillous trophoblast. The same group also demonstrated differential placental expression of these genes with gestation, with *PROK1* and *PROK1R,* increasing between 8 and 10 weeks of gestation while that of *VEGF* remained unaltered throughout the first trimester. They concluded that their data suggested complementary roles of PROK1 and VEGF in early pregnancy.

Taken together, these data is suggestive of distinct but complementary roles of these angiogenic factors where VEGF-A and its mitogenic receptor VEGFR2 may be mainly, but not exclusively, be responsible for mediating maternal angiogenesis and spiral arteriolar transformation in the placental bed while PROK1 and PROK1R exclusively mediate placental villous angiogenesis in the fetus. VEGF-A likely plays a critical role in the initial stages of villous capillary vasculogenesis and early angiogenesis (as evidenced by lethality in VEGF receptor and ligand knockout embryos) and may facilitate integration of invasive EVT cells into the maternal vascular system. PROK1 on the other hand is potentially involved in maintaining angiogenesis after VEGF-A has initiated vascular events in early pregnancy.

Kisspeptin could in addition to inhibition of metalloprotease activity, accomplish its anti-metastasic effects through the suppression of angiogenesis via factors such as VEGF and PROK1. Kisspeptin has been shown to reduce HUVEC (Human Umbilical Vein Endothelial Cells) migration, invasion and tube formation via Specificity Protein-1 (SP1) mediated suppression of VEGF expression [45]. Kisspeptin was subsequently shown to inhibit both new vessel sprouting and tube structure formation in HUVECs in a dose dependant manner [46]. Kisspeptin could therefore limit fetal invasion both by suppressing placental MMP9 expression as well as inhibiting placental bed angiogenesis via the suppression of VEGF A expression.

It should however be borne in mind that all the above findings are a description of the feto-maternal interphase at term. This is an acknowledged limitation of the study as a description of the molecular details of this interphase in early pregnancy would provide more information on feto-maternal dialogue. Nonetheless attempting such a study in early pregnancy could prove challenging firstly because true placental bed biopsies comprising decidua, extravillous trophoblast and myometrium might prove difficult to attain in early pregnancy and secondly studies of such a nature (ie involving placental bed sampling) might be limited by ethical considerations.

In summary our study has examined differential gene expression in both the fetal and maternal compartments of the feto-maternal interface. Figure 8 depicts a schematic representation of the anatomy of the feto-maternal interface (Fig. 8a) as well as the putative interactions of factors examined in this study at this interface (Fig. 8b). We have found high fetal expression of *Kiss1*, *GPR54* as well as *MMP9* which are all genes involved in invasion suggesting that the control of invasion capacity (at least involving these genes) may predominantly be exercised at the fetal level. Examination of genes involved in angiogenesis revealed high expression of *PROK1* and *PROK1R* ligand-receptor pair in the fetus and high expression of *VEGF-*A and *VEGFR2* ligand-receptor pair on the maternal side. VEGFR1, which is anti-VEGF, was highly expressed in the fetus. These findings may be suggestive of angiogenesis being prokineticin-driven in the placenta while maternal angiogenesis in the placental bed is mainly mediated by VEGF-A. Further studies will be required to explore the potential of this dual angiogenesis hypothesis. The findings could unveil the possible prophylactic use of VEGFR2 agonists in women with a predilection for preeclampsia and the potential use of PROK1 agonists in the treatment of fetuses with growth restriction. Having defined the differential gene expression profiles across the feto-maternal interface of healthy pregnancies, further studies need to compare these profiles with those of pathological pregnancies.
